# Obituary

**Published:** 1872-09-15

**Authors:** 


### OBITUARY.

Died, in Quito, on Sunday, July 28, 1872, after a protracted illness, of typhoid fever, Dr. Camilo Casares, in the 42d year of his age.

And thus suddenly, in the flower of his days, has passed from earth one who had already won an eminent reputation in his chosen profession, and whose future promised fair for science and surgery.

As a gentleman, he was always courteous and winning in his deportment. As a man, he was honorable, generous, tender-hearted, and dear to his friends. As a physician, he had but few superiors, and he stood pre-eminent as a surgeon. Laborious and faithful, he added to these virtues rare talent and a remarkable scientific intuition. He leaves vacant a place that cannot well be filled.

The indigent poor of Quito will not soon find another heart more gentle, or more generous to their sufferings. The circle of his profession will long remember his skillful aptitude and ready knowledge. His friends (and they were legion) will never forget the winning affability and genial brightness of his presence. To his family such a blow is irreparable. And his country has lost in him one of the noblest and most enlightened of her children. It will be difficult indeed to fill the position which he occupied with such signal skill in the University of Quito.

Talented, popular, and hopeful, it is one of

Heaven's strange mysteries that such a man should fall in the very tide of his usefulness, and the very May-day of his manhood.

Rest on, good friend. Sleep tranquilly, stout heart. Life's work was well and bravely done, and thou hast gone to thy reward.

A Friend.

